# Multi-Objective Optimization of the Mechanical Properties of 3D-Printed PLA: An Integrated Taguchi and NSGA-II Approach

**DOI:** 10.3390/polym18151821

**Published:** 2026-07-25

**Authors:** Zainab Hussein Mohsein, Diana Abed Alkareem Noori, Aseel Hamad Abed, Abbas Fadhil Ibrahim, Osama M. Irfan, Abdulrahman Mohammed Albar, Walid M. Shewakh

**Affiliations:** 1 College of Production Engineering and Metallurgy, University of Technology, Baghdad 10066, Iraq; 2 Department of Mechanical Engineering, College of Engineering, Qassim University, Buraydah 51452, Saudi Arabia; 3 Department of Industrial Engineering, Jazan University, Jazan 82817, Saudi Arabia

**Keywords:** fused deposition modeling, polylactic acid, Taguchi method, multi-objective optimization, NSGA-II, mechanical properties, additive manufacturing

## Abstract

Fused deposition modeling (FDM) of polylactic acid (PLA) is widely used, yet most parameter studies tune one mechanical property at a time and leave the conflicts between properties unresolved. This work treats three responses of FDM PLA together: ultimate tensile strength, flexural strength, and Shore D hardness. A Taguchi L9 orthogonal array varied infill density, raster angle, and layer thickness; signal-to-noise ratios and analysis of variance ranked the factors, linear regression linked the parameters to each response, and the NSGA-II algorithm mapped the trade-off surface between them. Layer thickness proved the leading factor for tensile and flexural strength, while hardness answered mainly to infill density; raster angle stayed weak for every response. The Pareto front showed that low infill favors tensile strength while high infill favors flexural strength and hardness, all at the finest layer setting, and these trends were converted into parameter guidelines for tensile-led, flexure-led, and balanced parts. The statistical limits of the screening design are stated openly, the flexural and hardness campaigns are flagged as provisional, and a replicated, standard-compliant confirmation study is set out as the next step.

## 1. Introduction

Additive manufacturing (AM), or 3D printing, builds geometrically complex parts directly from digital models, layer by layer, and has spread quickly across industry [1]. Among AM methods, fused deposition modeling (FDM), also called fused filament fabrication, is the most common route for thermoplastics because of its design freedom, low equipment and material cost, and broad material choice [2,3]. It serves rapid prototyping, low-volume production, and some structural and biomedical parts, which keeps it an active subject in manufacturing research [4].

Polylactic acid (PLA) is one of the most used FDM feedstocks. It comes from renewable sources such as corn starch, and it is biodegradable and biocompatible, which gives it an environmental edge over petroleum-based polymers [5]. PLA prints easily and typically gives a good surface finish, and while its dimensional accuracy is often acceptable, it is not guaranteed: shrinkage and accuracy vary with the process settings themselves. Mechanically, it remains limited, with lower ductility, modest impact resistance, and reduced thermal stability compared with engineering polymers [6,7]. Raising the mechanical performance of FDM PLA through careful choice of process parameters is therefore a real and useful problem [8].

Many studies have looked at how single FDM parameters change PLA behavior. Wang et al. [8] showed that printing angle, layer thickness, infill ratio, and nozzle temperature each affect tensile strength and dynamic properties. Tang et al. [9] reported that extrusion temperature and printing speed interact, with strength peaking inside a narrow temperature window. Le et al. [10] used the Taguchi method on thermoplastic polyurethane and found a 0.1 mm layer with a 45-degree fill to be best for tensile strength. Abdulridha et al. [11] combined a Taguchi L25 design with an artificial neural network for PLA and reported infill density as the leading variable for UTS and compressive strength. Moradi et al. [12] characterized FDM PLA across build orientation, infill density, and infill pattern, reporting strong anisotropy and a clear infill-pattern effect on tensile response; their work is a useful benchmark for the parameter ranges and the property trends discussed here.

Other work has targeted specific responses for various polymers. Oudah et al. [13] found infill density to dominate the compressive strength of ABS. Saleh et al. [14] linked PLA failures mainly to weak interlayer bonding under varying ambient temperature. Obaeed and Hamdan [15] optimized FDM parameters for the flexural strength of medical-grade PMMA. Abdulridha et al. [16] tuned PETG and identified platform temperature as the strongest factor for flexural strength and dimensional accuracy. Chacon et al. [17] gave a thorough study of how build direction, layer thickness, and feed rate change the tensile and flexural response of PLA, with the effect depending on the property in question. Sagias et al. [18] and Wankhede et al. [19] applied Taguchi analysis to ABS, and Al-Bdairy et al. [20] used a neural-network model to predict properties of rapid-prototyped parts. More recent Taguchi work on FDM PLA, from 2020 onward, points the same way while often using richer designs. Hikmat et al. [21] applied a Taguchi plan to raise the tensile strength of PLA parts and placed build orientation and infill among the strongest factors. Lokesh et al. [22] ran an L9 screening at constant temperature and again ranked layer thickness first for tensile response. Larger arrays are common where budgets allow: Singh et al. [23] used an L27 for PLA tensile behavior across build orientations, Ahmed et al. [24] ran an L18 with confirmation specimens and fracture-surface imaging, and Gultekin and Ozdemir [25] recently paired an L27 with a hybrid regression model for the flexural strength of PLA. The pull toward L27 in this literature reflects the low material and machine cost of PLA, a point taken up in Section 2.5 when the L9 used here is justified.

PLA deserves this attention because its practical advantages are hard to match. It prints at low temperature without a heated chamber, warps far less than ABS, comes from renewable feedstocks, is biodegradable and biocompatible, and costs little per part, which is why it carries so much of the consumer, educational, and laboratory FDM workload. Getting more out of such a material through parameter choice alone, with no change to hardware or feedstock, is the cheapest performance upgrade available to a practitioner. Two gaps remain in how that choice is usually made. First, most studies treat one response at a time, so they cannot resolve the conflicts that appear when several properties matter at once. Second, the few that optimize tend to use single-objective or rule-based methods. We are explicit that neither the Taguchi method nor evolutionary multi-objective search is new and that the two have been paired before in the FDM literature, including some works cited above. The contribution here is therefore not the tooling. It is the combination of three things in one consistent dataset: an integrated, end-to-end workflow that ties screening, regression, and a Pareto search to concrete application guidelines; a careful reading of two effects that screening data of this kind make easy to get wrong (the sign of the infill effect on flexural strength and the cause of the hardness trend); and an unusually plain accounting of the statistical limits, with the flexural and hardness results flagged as provisional rather than presented as settled. For a high-consumption material such as PLA, where vendors publish generic profiles, this kind of property-against-property trade-off map and the honest limits around it are the part a practitioner cannot read off a spool label. Put directly, the gap this study fills is the absence, for these three widely used properties measured on one printer, one filament batch, and one consistent protocol, of a single trade-off map that tells a practitioner what is gained and what is given up as the parameters move. What has not been done before, to our knowledge, is pairing such a map with an equally explicit statement of which parts of it are firm and which are provisional. For industry, the contribution is immediate: the guidelines in Section Application Guidelines convert the front into three ready-to-use settings for tensile-led, flexure-led, and balanced parts. The results also differ from parts of the existing record in one concrete way: higher infill raised flexural strength here, where several earlier reports suggest a decline, a difference this paper flags and schedules for confirmation rather than glosses over.

This work evaluates layer thickness, infill density, and raster angle against UTS, flexural strength, and Shore D hardness. A Taguchi L9 array sets the experiments, ANOVA quantifies each factor’s share of the variation, linear regression captures the parameter–property relations, and NSGA-II maps the trade-off surface for application-driven selection. The limits of the L9 analysis are stated throughout, and a validation path is given.

Table 1 sets this study against representative recent studies that used L9, L18, and L27 arrays and response-surface methods on FDM parts. Richer arrays and comparative designs exist, but none of the listed works maps the three-way trade-off among tensile strength, flexural strength, and hardness on one consistent dataset or converts that map into application guidelines.

## 2. Materials and Methods

**Figure 1:**
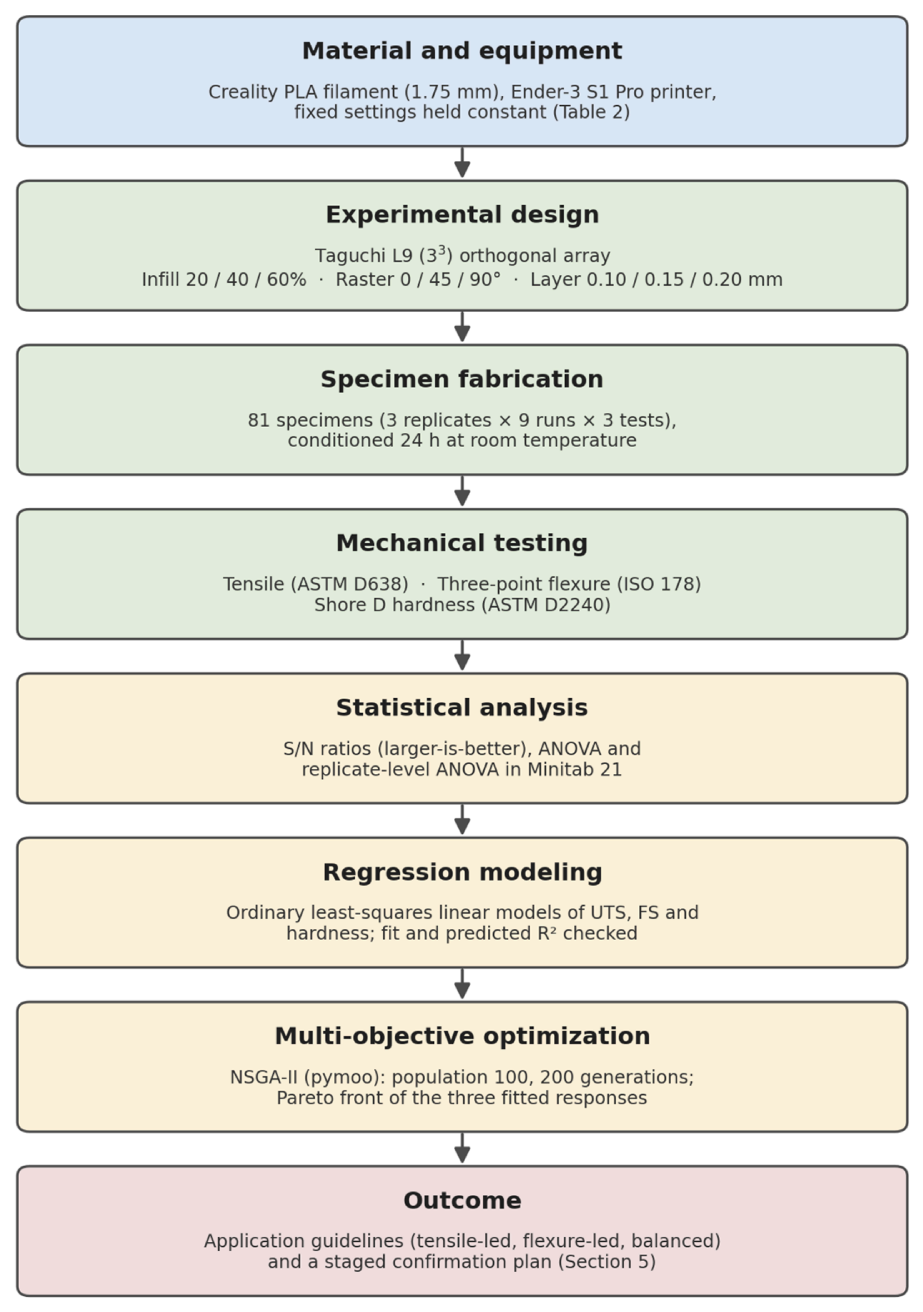
Workflow of this study.

Figure 1 summarizes the workflow of this study, from the material and fixed equipment settings; through the Taguchi design, specimen fabrication, mechanical testing, statistical analysis, and regression modeling; to the NSGA-II search; and ending in the application guidelines and the staged confirmation plan. Each stage is detailed in the subsections below.

### 2.1. Material

Commercial PLA filament of nominal diameter 1.75 mm (tolerance plus or minus 0.05 mm) was supplied by Creality (Shenzhen, China). The manufacturer lists a glass transition temperature near 60 °C, a melting range of 150 to 160 °C, a recommended extrusion range of 195 to 220 °C, and a density of 1.24 g/cm^3^. The whole study used a single red filament from one batch. Pigment can shift the tensile, flexural, and impact response of PLA by amounts that are not always small [27], so holding color and batch fixed keeps it from confounding the parameter comparisons rather than assuming the effect away. To limit moisture uptake, which weakens interlayer bonding and surface quality, spools were kept sealed at 23 plus or minus 2 °C and below 40% relative humidity for the whole study [28]. The supplier specifications of the filament are summarized in Table 2.

### 2.2. Equipment and Fixed Parameters

**Figure 2:**
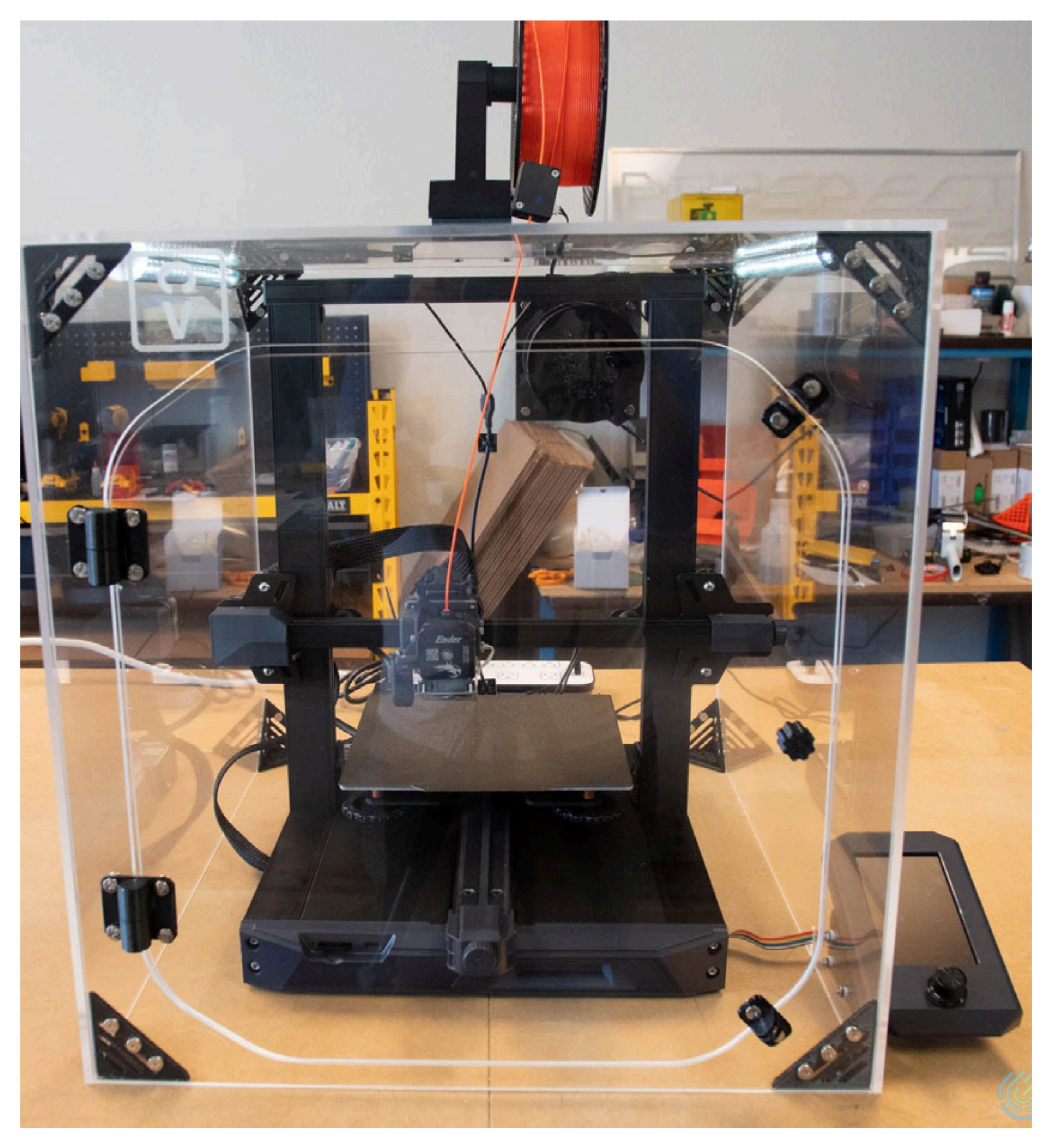
Photograph of the FDM printing setup (Creality Ender-3 S1 Pro) used to fabricate all specimens.

Specimens were printed on a Creality Ender-3 S1 Pro (Shenzhen Creality 3D Technology Co., Ltd., Shenzhen, China) with a 0.4 mm brass nozzle in a room held at 23 plus or minus 2 °C and 35 plus or minus 5% relative humidity. Fixed parameters, chosen from calibration trials and the literature to avoid warping, delamination, and stringing, are listed in Table 3. A cubic infill was used because it gives near-isotropic in-plane behavior and reduces directional bias. The 50 mm/s print speed was set because slower deposition lengthens thermal contact between layers and improves chain diffusion and adhesion [29,30]. The cooling fan was held at full speed after the first layer to control sagging and surface quality. That setting works against interlayer healing, since it cools the fresh interface quickly, so the layer-thickness effect discussed in Section 3.7 is best read as the net of deposition heat and active cooling rather than of layer thickness alone. The remaining slicer settings, including the wall count and the top and bottom layer counts, followed a single stored PLA profile that was left unchanged across all 81 specimens; slicing used Creality Print version 4. The printing setup is shown in Figure 2.

### 2.3. Specimen Design and Fabrication

**Figure 3:**
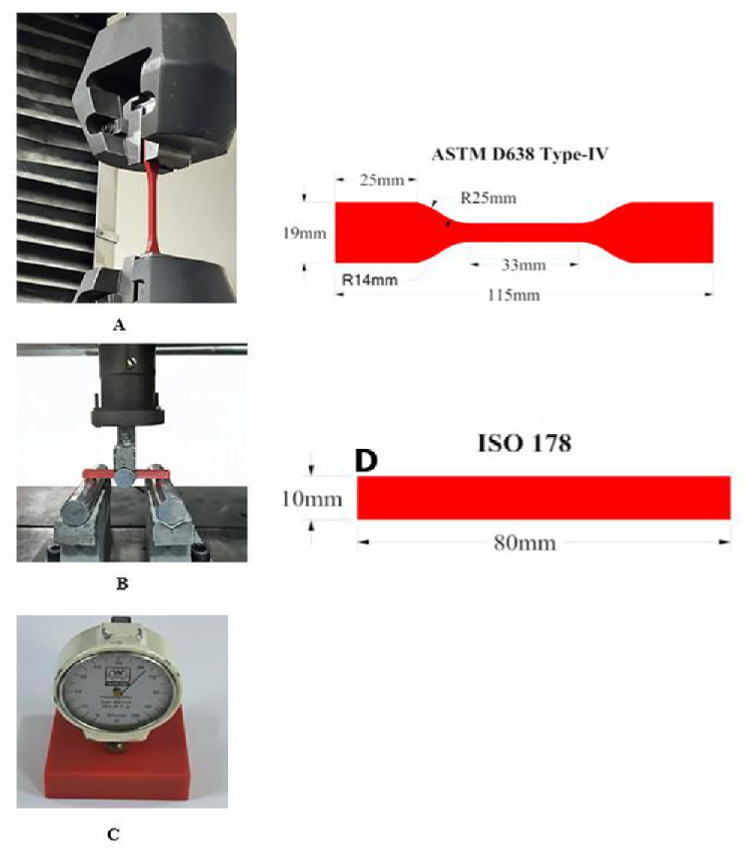
Mechanical testing equipment and specimen configurations (dimensions in mm): (**A**) universal testing machine used for the tensile tests; (**B**) three-point bending setup; (**C**) Shore D durometer; (**D**) specimen geometries. The hardness blocks share the flexural bar dimensions of 80 × 10 × 4 mm.

Specimens were designed in SolidWorks 2020 (Dassault Systemes, Velizy-Villacoublay, France) to the relevant standards. Tensile coupons followed ASTM D638 Type IV [32], with a 33 mm narrow-section length, a 25 mm gauge length, 6 mm gauge width, 4 mm thickness, and 115 mm overall length. Flexural bars followed ISO 178 [33] at 80 by 10 by 4 mm. Hardness blocks were 80 by 10 by 4 mm, with flat parallel faces. Three replicates of each type were printed per condition for 81 specimens across the nine runs. All specimens were held at room temperature for 24 h before testing to relieve residual thermal stress [31]. Figure 3 shows the testing setup and specimen geometries.

### 2.4. Mechanical Testing

#### 2.4.1. Tensile

Uniaxial tension followed ASTM D638 [32] on a PC-controlled electromechanical universal testing machine (model WDW-200E, Jinan Testing Equipment IE Corporation, Jinan, China; capacity 200 kN), shown in Figure 3A, fitted with a calibrated 20 kN load cell (Class 1 per ISO 7500-1 [34]). Type IV coupons were pulled at 5 mm/min with a 5 N preload, a 25 mm gauge length, and acquisition at 10 Hz. Raster angle (0, 45, and 90 degrees; Table 4) was set relative to the loading axis, so 0 degrees lays the roads parallel to the pull. Engineering stress was the instantaneous load over the original cross-section, and engineering strain was the crosshead displacement over the gauge length; UTS is the highest stress each coupon reached, and every value in Table 5 is the mean of three replicates. Only UTS is tabulated here. The elastic modulus, 0.2% offset yield strength, and elongation at break are now reported per condition in Table 6 (Section Additional Tensile Properties (Replicate-Level)) as mean plus or minus standard deviation from the three replicates. Because strain here comes from crosshead travel, which carries machine compliance, the moduli are given as apparent values, useful for ranking the conditions rather than as absolute material moduli; an extensometer-based measurement of the absolute modulus is kept in the future work. One point is worth stating plainly. Peak tensile loads sat near 0.9 kN, about 4.3% of the 20 kN cell capacity. That level sits below the 20% of full scale at which Class 1 is most commonly verified under ISO 7500-1 [34], so unless the cell’s calibration certificate confirms Class 1 down to this load, the UTS values should carry the same load-cell caveat raised for the flexural data. The early portion of each recorded trace shows the small step pattern this can produce, and we return to it in Section 3.8.

**Figure 4:**
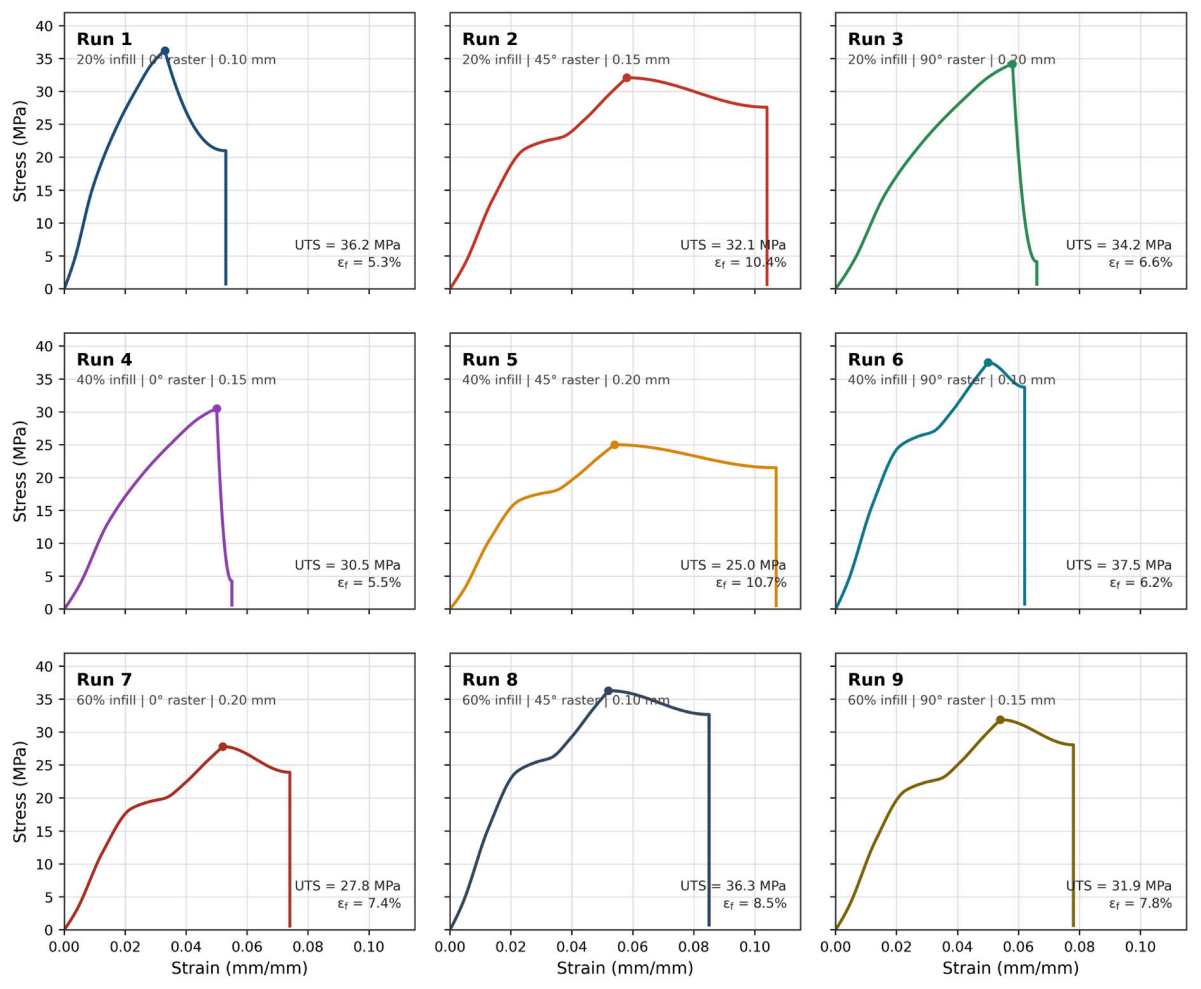
Schematic, illustrative tensile stress–strain curves for the nine L9 conditions, one panel per run. Panels are ordered left to right and top to bottom as runs 1 to 9 of Table 5, and each panel is titled with its run number, infill density, raster angle, and layer thickness.

Figure 4 gives a schematic stress–strain curve for each of the nine runs; these are illustrative reconstructions, not raw instrument traces. The peak of each curve is set to that condition’s mean UTS from Table 5, while the curve shape and the strain at break are taken from the recorded traces. They are shown to convey the form of the response and the spread between conditions and should not be read as measured curves. Strain here is taken from crosshead travel rather than an extensometer, so it carries some machine compliance; the curves are therefore best read for the shape of the response and the spread between conditions, not as a route to an exact elastic modulus.

The nine curves share the form expected of FDM PLA. A short toe gives way to a near-linear region, the slope then rolls into a yield zone, the load tops out, and the coupon fails soon after. The three runs printed at 0.10 mm (runs 1, 6, and 8) reach the highest peaks, near 36 to 37.5 MPa, which fits the leading role that layer thickness takes in the later analysis. Strain at break is noisier and harder to pin down. A few of the thicker-layer and 45-degree runs, runs 2 and 5 for instance, drew out past 10% before breaking, while the thinnest and stiffest coupons tended to part earlier, closer to 5 to 6%. We read this as a soft trade between strength and stretch rather than a firm law, since one trace per condition cannot tell a real trend from ordinary scatter. The replicate-level curves with error bands sit in the follow-up work of Section 5.

#### 2.4.2. Three-Point Flexure

Flexure used the same WDW-200E universal testing machine as in Section 2.4.1 (the bending setup is shown in Figure 3B) in three-point bending per ISO 178 [33], with a 64 mm span (span-to-depth 16:1), 5 mm support and nose radii, 1 N preload, 2 mm/min crosshead speed, and 10 Hz acquisition. Flexural strength was computed as 3FL over 2bd-squared, with L = 64 mm, b = 10 mm, and d = 4 mm [33]. For consistency, raster angle was defined for the bending bars relative to the long (span) axis, so that 0 degrees places roads along the span and 90 degrees places them across it; the same convention was kept for the hardness blocks. This definition is stated explicitly because a given nominal raster level is not the same physical orientation across the three load modes. Important caveat: the WDW-200E is a 200 kN-class frame. The flexural failure loads here (about 50 to 100 N) fall near 0.025 to 0.05% of that capacity, far below the verified range of a Class 1 system under ISO 7500-1 [34]. The coarse, repeated flexural values (30, 45, 60 MPa) are therefore best read as a load-cell quantization artifact rather than a true material discretization. The flexural results are treated as provisional throughout, and Section 3.8 and the Future Work list call for a repeat on a load cell of about 1 kN or smaller.

#### 2.4.3. Shore D Hardness

Surface hardness followed ASTM D2240 [35] with a Shore D durometer (AMSLER, Schaffhausen, Switzerland; the instrument is shown in Figure 3C). Readings were taken 15 s after firm, perpendicular contact, three locations per specimen at least 6 mm apart. The reported value for each condition is the mean of nine readings (three specimens by three locations). One limitation must be stated up front: ASTM D2240 calls for a specimen at least 6 mm thick, whereas the hardness blocks here were 4 mm. A thin coupon on a rigid anvil can inflate Shore D readings and can blur the separation of the infill-density effect from a support effect. The hardness values are therefore reported as provisional and, like the flexural data, are flagged for re-testing with compliant (≥6 mm) coupons. A second point limits the weight these numbers carry. The whole hardness spread is only about 6.5 Shore D units, close to the repeatability of a hand durometer on printed parts, so the high regression R-squared for hardness reflects a tight fit to a narrow range rather than strong certainty about the underlying values.

### 2.5. Taguchi Design

A Taguchi L9 (3^3) array screened three factors at three levels each (Table 4), cutting the full factorial from 27 to 9 runs while keeping the design balanced for main-effect estimation [36]. The three factors were chosen because the literature most often names them as the drivers of PLA FDM performance [8,17,18]. Infill of 20 to 60% spans practical values; raster of 0 to 90 degrees covers the in-plane range; layer thickness of 0.10 to 0.20 mm covers the fine-to-coarse range for a 0.4 mm nozzle [29]. The choice of the L9 deserves a fuller justification than the earlier version gave. The L9 was selected as a screening array whose job is to rank main effects, and for that job, it is statistically adequate: three factors at three levels consume six degrees of freedom, the array supplies eight, and the design stays balanced and orthogonal, so each level mean rests on an equal share of the runs [36]. Its known costs are equally real: it cannot separate two-factor interactions from main effects, and an analysis on condition means leaves only two error degrees of freedom. Because every condition was printed and tested in triplicate for each response, the campaign still contains 81 physical specimens, and this replicate structure supplies the pure-error information the array itself lacks (Section 3.2). We agree that, with the low material and machine cost of PLA, a larger array carries little extra burden and that much of the recent PLA literature has moved to L27 for exactly that reason [23,25]; an L27 (the full 3^3) or a replicated L18 would add the interaction information an L9 cannot give, and that step is set out as the validation route in Section 3.8 and Section 5. The staged approach used here, a small screening array first and a larger confirmatory design second, is standard sequential experimentation [37], not a shortcut. The Taguchi design, signal-to-noise analysis, and ANOVA were carried out in Minitab 21.

### 2.6. Signal-to-Noise Ratio

Because higher values are desirable for all three responses, the larger-is-better criterion was used. The signal-to-noise (S/N) ratio for each condition is

**Figure 5:**
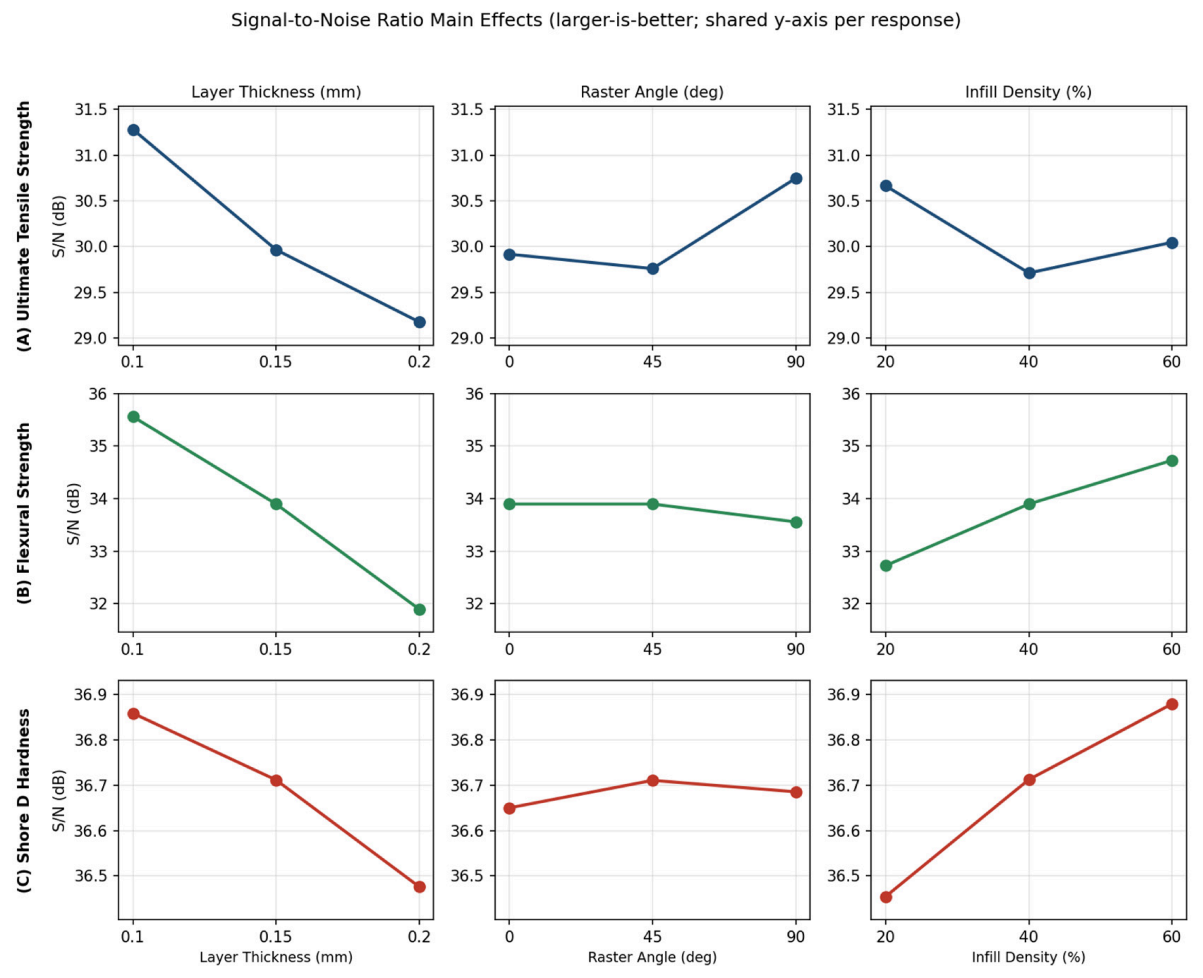
Signal-to-noise main effects for (**A**) UTS, (**B**) flexural strength, and (**C**) Shore D hardness; each row shares a common vertical axis.

*S*/*N* = −10 *log*_10_ [(1/*n*) *Σ_i_* (1/*y_i_*^2^)]

where y_i_ are the replicate responses in a condition and n is the number of replicates. One limit of how the ratio is computed here should be flagged: because the S/N was taken on the condition means rather than on the three replicates, it reduces to twenty times the base-ten logarithm of the mean and so carries no information about within-condition scatter. It therefore ranks mean performance, not robustness. The replicate scatter that the larger-is-better criterion is meant to reward is reported separately as the standard deviations in Table 6, and a replicate-level S/N is part of the planned follow-up. The S/N main effects are reported per response in Figure 5, and the level means and their ranges are tabulated in Table 7 so the ranking can be read directly rather than only described in words.

### 2.7. Analysis of Variance Procedure

ANOVA quantified each factor’s share of the total variation at the 0.05 level. The contribution of a factor is its sum of squares over the total sum of squares [37]. One point must be stated clearly: the analysis here uses the nine condition means, which leaves only two error degrees of freedom. This is enough to rank contributions but too little to confirm formal significance. The within-condition replicate data should be analyzed at the raw level to obtain a proper pure-error estimate and valid F-tests; the three-replicate measurements were recorded for all 81 specimens, and that raw-level analysis is the first item of the planned follow-up work (Section 5). A first instalment of that raw-level analysis is now included. A replicate-level ANOVA on the elastic-modulus data (27 specimens) is given in Section 3.2; with 20 error degrees of freedom, it supplies the pure-error term and valid F-tests the L9-means analysis could not, and it provisionally supports layer thickness (*p* < 0.001) and infill density (*p* = 0.002) as significant, while raster angle is not (*p* = 0.157).

### 2.8. Regression

Linear models of the form Y = b0 + b1(LT) + b2(RA) + b3(ID) were fitted by least squares, with LT in mm, RA in degrees, and ID in percent. Fit was judged by R-squared, adjusted R-squared, the standard error, and residual plots for linearity, constant variance, and independence [38]. Two points about the modeling pipeline are stated plainly to prevent misreading. First, every response value entering the regression is a measured experimental mean from Table 5; no synthetic or simulated data were generated at any stage of this study, and no noise model was needed because none would have had anything to stand in for. Second, the models were fitted by ordinary least squares in the general regression module of Minitab, which is separate from its Taguchi design module; the Taguchi module served only for the array construction and the S/N and ANOVA analyses. The regression is therefore a standard fit to measured data, not an output of the Taguchi routine itself, and the title of the paper describes the design and the optimizer, both of which are used exactly as named. With nine points and four coefficients, five residual degrees of freedom remain, so the models are not saturated. One modeling caveat is acknowledged at the outset: the raster effect is weak and non-monotonic in the S/N and ANOVA results, so a single linear slope cannot describe it well. We keep the linear raster term only for completeness, do not interpret its sign, and do not prescribe a raster angle from it (see Section 3.3 and Section 3.8).

### 2.9. Multi-Objective Optimization with NSGA-II

NSGA-II [39] searched for parameter sets that satisfy the three objectives at once. It was run in Python 3.11 (pymoo, version 0.6.1) with a population of 100, 200 generations, simulated binary crossover, and polynomial mutation, over the bounds 20 to 60% infill, 0 to 90 degrees raster, and 0.10 to 0.20 mm layer. The procedure was as follows. (1) Decision variables: the three process parameters, each bounded as above and treated as continuous within range. (2) Objectives: maximize the three regression models of Section 3.3; for the minimization engine each was negated. (3) Initial population of 100 sampled across the box. (4) Each individual evaluated on the three surrogates. (5) Fast non-dominated sorting ranks individuals into Pareto fronts. (6) Crowding distance measures spacing within a front to preserve diversity. (7) Binary tournament selection on rank then crowding distance. (8) Simulated binary crossover (eta = 15) and polynomial mutation (eta = 20) make offspring. (9) Parents and offspring are merged and truncated back to 100 by rank and crowding (elitism). (10) Steps 4 to 9 repeat for 200 generations; convergence was tracked by hypervolume (Section 3.6).

We note plainly that, because the surrogates are linear, the optimum of each objective lies at a corner of the box, and the Pareto set lies on its boundary; the same trade-off could be found analytically. NSGA-II is used here as a convenient, reusable solver that will also handle the nonlinear surrogates planned for future work and as a way to sample the trade-off curve densely. For the linear surrogates used here, the solver adds little beyond a dense sampling of a line segment, and we do not claim otherwise. Its role in this paper is the reusable stage that the nonlinear and interaction models planned in Section 5 will need, where a corner solution no longer holds. Other multi-objective methods such as MOPSO [40] or hybrid PSO-based evolutionary schemes [41] would serve the same role. The optimizer evaluates only these fitted response models; it neither creates nor consumes any synthetic dataset beyond them, and the complete dataset behind the models is provided as Supplementary Material (Table S1), and the bounds and objective functions are stated in full in Section 2.9 and Section 3.3. Because the front edges fall at untested corner combinations, the predicted optima are not accepted as results until the confirmation runs of Section 5 are completed.

### 2.10. Repeatability and Quality Control

Repeatability was controlled in three ways. First, three specimens were printed and tested per condition for each response, and Shore D was sampled at three locations per specimen, so each hardness mean rests on nine readings. Second, the build environment was held within narrow bounds (23 ± 2 °C, 35 ± 5% RH), filament was kept dry and sealed, and the machine was calibrated before each batch, since drift in any of these is a common source of run-to-run scatter in FDM. Third, fabrication order was kept consistent, and specimens rested 24 h before testing. The quantitative side of repeatability, namely per-condition standard deviations and coefficients of variation from the replicate set, is the subject of the raw-level analysis committed to in Section 5; it is not estimated from the condition means reported here.

## 3. Results and Discussion

### 3.1. Experimental Data

Table 5 lists the L9 matrix and the measured means. Values are reported to one decimal place throughout for consistent precision. Per-condition tolerances are reported where the underlying replicate records allow: Table 6 gives mean plus or minus standard deviation for the replicate-level tensile properties. For UTS, flexural strength, and hardness, the raw per-specimen records are provided as Supplementary Material (Table S1); the flexural values, in particular, are quantized by the over-range load cell (Section 2.4.2), so a standard deviation computed from them would understate the true uncertainty. UTS ranged from 25.0 to 37.5 MPa, flexural strength ranged from 30 to 60 MPa, and hardness ranged from 64.5 to 71.0 Shore D, so the chosen ranges do move the responses. The three runs at 0.10 mm (runs 1, 6, 8) gave the highest UTS (36.2, 37.5, 36.3 MPa) and the highest hardness (68.0, 70.0, 71.0 Shore D), an early sign that layer thickness leads.

#### Additional Tensile Properties (Replicate-Level)

The same tensile records give three more properties beyond UTS: the apparent elastic modulus, the 0.2% offset yield strength, and the elongation at break. These are reported as mean plus or minus standard deviation from the three replicates of each condition (Table 6), which also supplies the per-condition tensile scatter that the earlier version had deferred.

The stiffness and yield results echo the UTS picture. The three 0.10 mm conditions (runs 1, 6, 8) give the highest modulus (3150 to 3280 MPa) and the highest yield (34.5 to 35.8 MPa), while the coarse-layer, mid-infill run 5 (40%, 45 deg, 0.20 mm) is the softest and weakest (2210 MPa, 23.5 MPa) and also the most ductile (9.5%). Elongation at break moves the other way to strength, rising as the layer coarsens, which fits a soft strength-against-stretch trade rather than a strict law. Because the moduli are crosshead-derived, they are read for ranking, not as absolute values; the standard deviations are small next to the spread between conditions, so the ordering is stable.

The S/N main effects in Figure 5 confirm the picture. In the revised figure, the three panels for each response share a common vertical scale, so the apparent slopes are now directly comparable and small differences are not visually magnified. Layer thickness gives the steepest slope for UTS and flexural strength, so it is the strongest factor for those two. Infill density rises steadily for flexural strength and for hardness. Raster angle is weak and non-monotonic for every response; for flexural strength, its three-level means are essentially equal, so it carries almost no main effect here.

### 3.2. Analysis of Variance

Table 8 gives the ANOVA on the condition means. Layer thickness is the largest contributor to UTS (65.8%) and flexural strength (66.7%). Hardness is governed mainly by infill density (52.7%), with a strong second contribution from layer thickness (42.8%), while raster angle adds only 1.2%. This corrects a common assumption that raster orientation should drive a surface property; for Shore D, which probes the near-surface bulk, the density of material under the indenter (set by infill and by layer compaction) matters more than in-plane road direction.

A statistical caution stands. With only two error degrees of freedom, no factor reaches the 0.05 threshold, even where the contribution is large (for example, layer thickness for UTS at *p* = 0.104 and infill for hardness at *p* = 0.059). These *p*-values reflect the thin error estimate of an unreplicated L9 analysis, not the absence of a physical effect. The contributions should be read as engineering trends. A replicated design (for example, L18 with replication or response surface methodology with center points) is needed to fix significance [37].

To replace the thin two-degree-of-freedom error of the L9-means analysis, the three tensile replicates were analyzed at the raw level for the elastic modulus. Table 9 gives this replicate-level ANOVA. With 27 specimens and 20 error degrees of freedom, the F-tests are now valid: layer thickness is highly significant (*p* < 0.001), infill density is significant (*p* = 0.002), and raster angle does not reach significance (*p* = 0.157). This gives a provisional, replicate-backed indication of the layer-thickness and infill effects that the means-based tables could only rank; it becomes a firm confirmation once the underlying raw data are deposited and independently checkable.

### 3.3. Regression Models

The least-squares fits are given in full below, with LT in mm, RA in degrees, and ID in percent. The earlier version named “the following models” without printing them; the equations, their coefficients, and the fit statistics are now stated explicitly (Table 10) so the optimization objectives are reproducible.

*UTS* (*MPa*) = 44.54 − 76.43·*LT* + 0.0336·*RA* − 0.0549·*ID*

*FS* (*MPa*) = 70.00 − 200.0·*LT* + 0.000·*RA* + 0.250·*ID*

*HD* (*Shore D*) = 69.28 − 30.00·*LT* + 0.0037·*RA* + 0.0833·*ID*

The large negative layer-thickness coefficients for UTS (−76.4) and flexural strength (−200.0) confirm its leading role, in line with the ANOVA. Two points about these regression results deserve note. First, the flexural model does not have a perfect fit; its honest R-squared is 0.833, with a 5.48 MPa standard error, and the residual degrees of freedom are five, not zero. A claim of R-squared equal to one for this response is not supported by the data. Second, the infill coefficient for flexural strength is positive (+0.25), so higher infill raises flexural strength rather than lowering it. The level means make this plain: 45, 50, and 55 MPa at 20, 40, and 60% infill. There is therefore no need for a residual-stress mechanism to explain a fall that does not occur. The flexural model is still best used inside the studied range and as an interpolation tool, and confirmation runs with added points are advisable [37].

**Figure 6:**
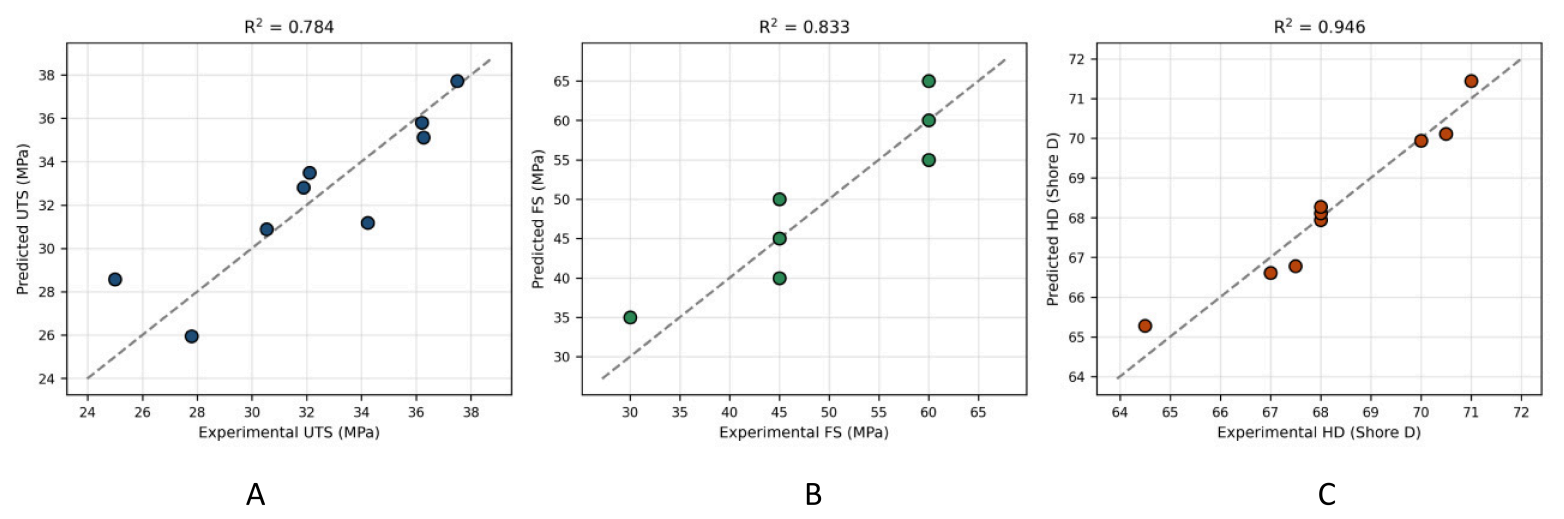
Predicted versus experimental values for (**A**) UTS, (**B**) flexural strength, and (**C**) Shore D hardness. Closer clustering about the dashed line of equality indicates a better fit.

The raster coefficients are small for all three responses, and for flexural strength, the term is effectively zero. Because the raster effect is weak and non-monotonic, a single linear slope is a poor descriptor, and we do not prescribe a raster angle from this dataset. The same caution applies to the infill term for UTS. Its main effect is non-monotonic, dipping at 40% infill and recovering at 60% (Figure 5A), so the small negative linear infill coefficient for UTS is not a real downward slope and is not interpreted as one, exactly as for raster. For hardness, the high R-squared (0.946) should also be read with care. It is a tight fit over a narrow band of about 6.5 Shore D units, near the repeatability of a hand durometer, so it reflects low scatter across a small range rather than strong predictive power. This is a genuine limitation of the linear form rather than a property of the material; a curved or categorical raster term, fitted on a replicated design, would describe it better. As a further check on predictive reliability, leave-one-out cross-validation was computed from the nine tabulated condition means. The predicted R-squared values come to about 0.13 for UTS, 0.35 for flexural strength, and 0.78 for hardness. The gap between fitted and predicted values is the honest price of nine points and four coefficients: the models describe the studied conditions adequately but should not be trusted far from them, which is one more reason the optimization endpoints are treated as hypotheses for confirmation rather than as results. This cross-validation is also internal to the nine points it was computed from; a genuinely external check, in which the fitted models are scored against specimens they never saw, is possible only with the confirmation runs of Section 3.8 and is committed there. Improving the models with interaction or curvature terms is not statistically defensible on nine points; that improvement belongs to the replicated design planned in Section 5. Figure 6 shows predicted against measured values; points sit close to the line of equality for UTS and hardness and within the wider scatter expected for the flexural model.

### 3.4. Residual Diagnostics

**Figure 7:**
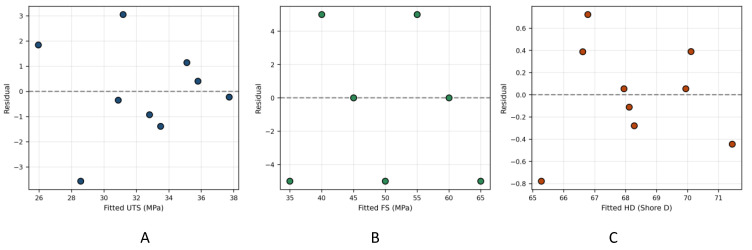
Residuals versus fitted values for (**A**) UTS, (**B**) flexural strength, and (**C**) Shore D hardness.

Figure 7 plots residuals against fitted values. Figure 5 and Figure 6 are kept because they answer different questions: Figure 6 (parity) shows overall goodness of fit and bias against the line of equality, while Figure 7 (residual-versus-fitted) tests the modeling assumptions of constant variance and independence and exposes any structure the parity plot hides. They are not redundant, even though both use the fitted values. For UTS and hardness, the residuals scatter around zero, with no clear curve or funnel shape, so linearity and roughly constant variance hold within the studied range [38]. The flexural residuals are larger and take a small set of values, a direct result of the quantized flexural readings; as set out in Section 2.4.2 and Section 3.8, that quantization is an artifact of an over-range load cell rather than a material feature, so finer flexural data with reported scatter are needed before firm mechanistic conclusions are drawn.

The largest single residual is Run 5 (40% infill, 45 deg, 0.20 mm), whose measured UTS of 25.0 MPa sits about 3 MPa below the model. This run pairs the coarsest layer (0.20 mm) with mid-range infill, the combination least favorable to interlayer healing, and it also lies at a design corner where the linear model extrapolates least comfortably. We flag it rather than discard it: with single condition means and no replicate spread, we cannot tell a true low point from ordinary scatter, which is one more reason the replicate-level re-analysis matters.

### 3.5. Multi-Objective Optimization

Single-objective tuning is not enough because the best settings for different properties pull apart. UTS is highest at low infill, while flexural strength and hardness climb with infill; layer thickness, by contrast, does not trade-off, since 0.10 mm helps all three at once and simply pins to its lowest level. NSGA-II was therefore used to map the remaining trade-off.

**Figure 8:**
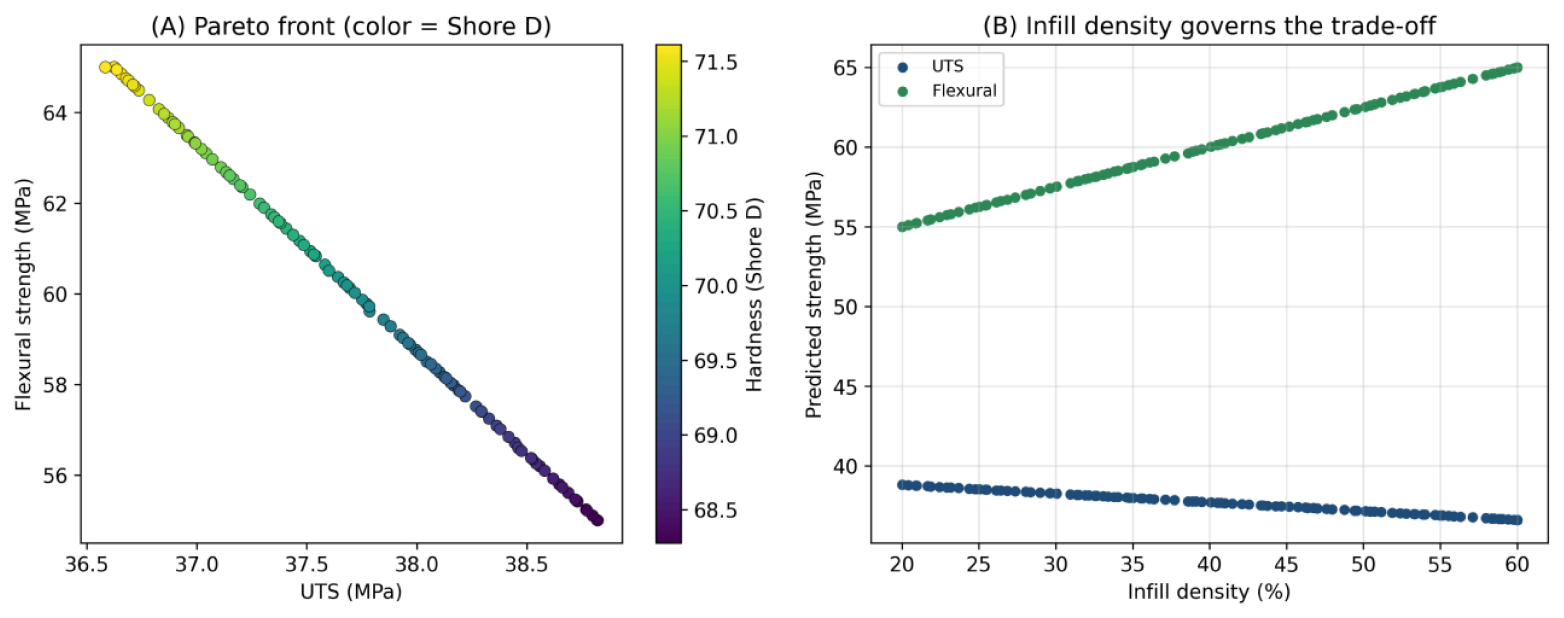
(**A**) Pareto front in the UTS–flexural plane, colored by Shore D hardness. (**B**) The trade-off is governed by infill density at fixed 0.10 mm layer thickness: tensile strength falls as infill rises, while flexural strength and hardness increase.

The search returned a clean, well-spread front of 100 non-dominated solutions. Every solution sits at the minimum layer thickness (0.10 mm), and the front is traced out almost entirely by infill density: low infill at the high-UTS end and high infill at the high-flexure and high-hardness end (Figure 8). Because the flexural axis rests on only three distinct, quantized values (Section 2.4.2), the flexural side of this front is provisional. The tensile axis is the firmer of the two, so the flexure-led endpoint should be read as a hypothesis to confirm rather than a result. Raster angle settled near 90 degrees because of the small positive linear coefficients for UTS and hardness, but since that effect is within the noise of this design, it should be read as a weak preference rather than a firm rule. Raster angle is also defined relative to a different physical axis for each of the three tests (Section 2.4.1, Section 2.4.2 and Section 2.4.3), so on the shared optimization axis, a single nominal raster level does not map to one fixed road orientation across the objectives; this is a further reason not to read a firm raster setting off the front. The predicted optima at the front edges (UTS near 38 to 39 MPa, flexural strength near 65 MPa) lie slightly above the measured maxima, which is expected for a model evaluated at untested corner combinations; Taguchi practice requires confirmation runs before such optima are accepted, so these endpoints are reported as predictions to be verified, not as achieved values (Section 5).

#### Application Guidelines

Three practical settings follow, all at 0.10 mm layer thickness, with raster angle treated as a secondary choice. Because the flexural and hardness data are provisional (Section 3.8), the flexure- and hardness-led guideline below should be confirmed on standard-compliant specimens before load-bearing use.

- **Tensile-led parts (about 20% infill):** highest tensile strength, with moderate flexural strength and hardness. Best where a single uniaxial load path dominates, for example, printed fasteners, tie rods and tension links, lightweight brackets loaded in pull, and snap hooks [42].
- **Flexure- and hardness-led parts (about 60% infill):** highest flexural strength and surface hardness, with somewhat lower tensile strength. Suited to parts that bend or take contact wear, such as cantilever beams and brackets, snap-fit lids, jig and fixture bodies, gear blanks, and bearing or wear pads [43,44].
- **Balanced parts (about 40% infill):** a reasonable compromise across all three properties for general structural use where no single load mode dominates, for example, housings, enclosures, and multi-purpose structural brackets that see mixed tension, bending, and surface contact.

### 3.6. Convergence and Population Size

**Figure 9:**
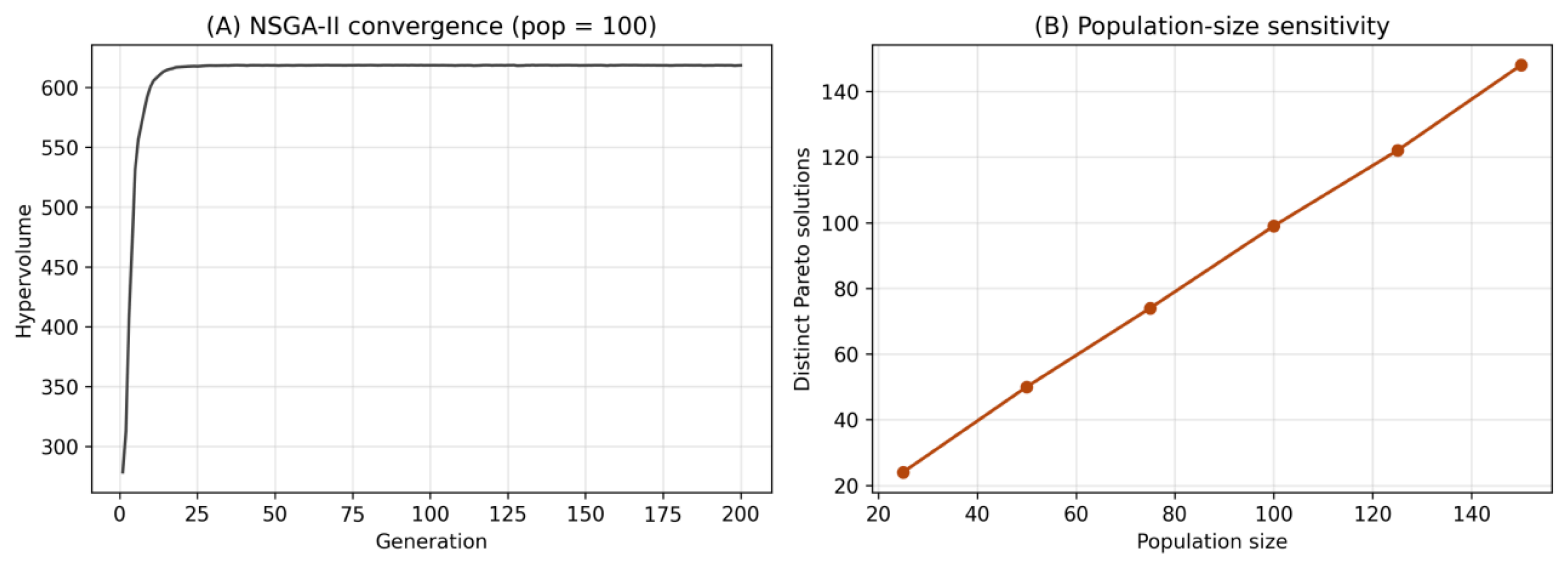
(**A**) Hypervolume against generation for the population of 100, showing stable convergence. (**B**) Distinct Pareto solutions against population size, rising roughly linearly across the tested range; a population of 100 was chosen as a cost-and-coverage compromise.

Figure 9 reports the search behavior. The hypervolume indicator rises and then flattens well before the 200th generation, which shows stable convergence without early stagnation. A sweep of population size from 25 to 150 shows the number of distinct front points rising roughly in step with the population across the whole tested range (Figure 9B), with no clear plateau by 150. A population of 100 was kept as a practical compromise between how densely the front is sampled and runtime, not because the returns flatten out. A comparison with alternative optimizers is fair to ask for, and for the linear surrogates used here, it can be settled analytically. A weighted-sum scalarization of three linear objectives is itself linear, so its optimum sits at a vertex of the design box for every weight vector, and sweeping the weights traces exactly the boundary segment NSGA-II returned; any correct solver, including MOPSO [40] or hybrid PSO schemes [41], must land on the same set, so differences reduce on this problem to how densely and evenly the segment is sampled. The more consequential methodological choice at this scale is the experimental design feeding the models, not the solver: Gao et al. [26] showed that Taguchi and response-surface designs can rank FDM factors differently on the same process. A formal solver benchmark becomes meaningful once nonlinear surrogates from the replicated design exist, and it is planned alongside them (Section 5). These results describe the solver working on smooth linear objectives and should not be read as evidence about the material itself.

### 3.7. Mechanisms

The leading role of layer thickness rests on three linked effects. Thinner layers keep the interface hotter during deposition, which lets polymer chains diffuse across the bond by reptation; they raise the bonded area per unit cross-section; and they lower the thermal gradient on cooling, which trims the residual stress that would otherwise cut apparent strength [45,46]. Together, these explain the large layer-thickness contribution to UTS and flexural strength.

The hardness trend, which a reviewer rightly asked us to explain, follows from the same picture rather than from anything unusual. Shore D measures resistance to indentation over a small near-surface volume, so it responds to how much solid PLA sits under the indenter tip. Raising infill from 20 to 60% puts more material directly beneath the surface and stiffens the shell-plus-infill region the tip pushes on, which raises the reading; this is the 52.7% infill contribution in Table 8. A thinner layer adds to the same effect by compacting the near-surface roads more tightly and leaving fewer and smaller inter-road voids, which is the 42.8% layer-thickness contribution. Raster angle barely moves hardness (1.2%) because the indenter samples through the layers rather than along a single road, so in-plane road direction is largely averaged out. The changes are therefore modest in size (about 64.5 to 71 Shore D) and consistent in direction, not erratic; the residual concern is the sub-standard 4 mm coupon thickness (Section 2.4.3), which may shift the absolute values upward and is addressed by the planned re-test.

Infill density raises flexural strength and hardness because more solid material under the loaded surface increases both bending resistance and resistance to indentation. The cubic infill spreads internal load fairly evenly, which keeps the bending response close to isotropic and limits coupling between the infill geometry and the outer shell [47]. The mild fall of tensile strength at higher infill seen in these data is weak and within the design noise, so we do not over-read it. Raster anisotropy is real in FDM parts, since the road-direction covalent bond far exceeds the weaker bond across roads [43]; in this particular dataset, however, the raster main effect did not separate from noise, and we report it as such rather than forcing an interpretation. Direct microstructural evidence, namely representative stress–strain curves and fracture-surface imaging by SEM or micro-CT to reveal raster-gap, neck-growth, and inter-bead voids of the kind reported for FDM PLA [12,48], is the most useful next step for tying these trends to mechanism and is listed in the Future Work.

### 3.8. Limitations and Validation Plan

First, the unreplicated L9 analysis on condition means carries only two error degrees of freedom, so its *p*-values rank rather than confirm; the replicate-level ANOVA of Table 9 begins to repair this for the tensile campaign. Second, the flexural readings are quantized by a load cell operated far below its verified range, so the flexural results, and everything downstream of them, including the flexure-led side of the Pareto front, are provisional. Third, the Shore D coupons were 4 mm thick against the 6 mm minimum of ASTM D2240 [35]; the planned re-test will use plied or thicker coupons that meet the standard. Fourth, the predicted optima at the front edges fall at untested corner combinations, and Taguchi practice requires confirmation runs before such optima are accepted. The validation plan is therefore concrete: print new specimens at the three guideline settings of Section Application Guidelines in triplicate, so that each optimum carries three repetitions, test them to the standards on appropriately ranged load cells, compare measured against predicted values as an external validation of the regression models, and image the fracture surfaces of at least the three optimum samples by SEM to tie the observed trends to mechanism [24].

## 4. Conclusions

An integrated Taguchi and NSGA-II workflow was applied to the multi-objective optimization of FDM PLA. The conclusions are grouped by what was found, how it is explained, what it yields in practice, and how firmly it stands.

**Dominant factors.** Layer thickness is the leading process parameter for tensile and flexural strength (65.8% and 66.7% of the variation), and a 0.10 mm layer gave the best result for all three properties through better thermal healing, larger bonded area, and lower residual stress [45]. Hardness is governed mainly by infill density (52.7%), with a strong layer-thickness contribution (42.8%), not by raster angle, which fits Shore D probing near-surface bulk material rather than in-plane road direction (Section 3.7). Raster angle itself has a weak, non-monotonic effect that did not separate from noise for any response, so no raster setting is prescribed from this dataset.

**Replicate-level support.** The replicate-level tensile properties (apparent modulus, 0.2% offset yield strength, elongation at break) are reported as mean plus or minus standard deviation for all nine conditions (Table 6). A replicate-level ANOVA on the modulus, with 20 error degrees of freedom, provisionally supports layer thickness (*p* < 0.001) and infill density (*p* = 0.002), while raster angle is not significant (Table 9).

**Trade-off and practical guidelines.** NSGA-II mapped a clean trade-off driven by infill density at a fixed 0.10 mm layer thickness: low infill for tensile-led parts, high infill for flexure- and hardness-led parts, and about 40% infill for balanced parts, converted into the three ready-to-use settings of Section Application Guidelines. Because the surrogates are linear, this front is also reachable analytically; the evolutionary solver is kept for convenience and for the nonlinear models planned next.

**Standing of the evidence.** Higher infill is associated with higher flexural strength in these data, which contrasts with the decline sometimes reported for FDM PLA; because the flexural readings are quantized, this is a provisional trend to be checked on standard-compliant specimens. More broadly, the L9 means analysis leaves two error degrees of freedom, and the flexural and hardness campaigns carry the instrument-related caveats collected in Section 3.8. Replicated, standard-compliant designs are needed to confirm significance, with per-condition standard deviations reported [37].

## 5. Future Work

- Print and test confirmation specimens at the three guideline settings of Section Application Guidelines on standard-compliant coupons and appropriately ranged load cells and compare measured against predicted values, with three repetitions per setting, as an external validation of the fitted models.
- Add nozzle temperature, print speed, and shell count in a replicated response surface design and fit nonlinear or interaction terms for the non-monotonic raster effect.
- Image fracture surfaces (SEM, micro-CT) of at least the three optimum-setting samples to test the proposed bonding and residual-stress mechanisms directly.
- Extend the workflow to ABS, PETG, PC, TPU, and fiber-reinforced PLA and test optimized settings on real components, not just coupons.

## Figures and Tables

**Table 1:** This study set against representative recent Taguchi and response-surface optimization studies of FDM parts [22,23,24,25,26].

Study	Design	Response(s)	Approach	Relation to This Work
Lokesh et al., 2022 [22]	L9	Tensile properties of PLA	S/N ranking at constant printing temperature	Single response; no optimizer and no trade-off map
Singh et al., 2022 [23]	L27	Tensile behavior of PLA across build orientations	Full 3^3 factorial with interaction resolution	Richer array, but one response and no multi-objective search
Ahmed et al., 2023 [24]	L18	Tensile strength of PLA	Taguchi design with confirmation specimens and fracture imaging	Validation practice that this study adopts in its plan (Section 3.8 and Section 5)
Gao et al., 2022 [26]	Taguchi and RSM	Mechanical strengths of FDM parts	Comparative Taguchi versus response-surface designs	Shows the choice of design can change factor rankings; motivates the replicated RSM step planned here
Gultekin and Ozdemir, 2026 [25]	L27	Flexural strength of PLA	Hybrid regression model fitted on the array	Regression on an array as here, but one response and no Pareto front
This study	L9 with an 81-specimen replicate structure	UTS, flexural strength, and Shore D hardness together	OLS regression, NSGA-II Pareto front, and application guidelines	Three-way trade-off map on one consistent dataset, with a staged confirmation plan

**Table 2:** Supplier specifications of the PLA filament, as listed by the manufacturer for the batch used.

Property	Specification	Note
Manufacturer	Creality (Shenzhen, China)	Commercial supplier
Material, color, batch	PLA, red, single batch	Held fixed to avoid pigment confounding [27]
Filament diameter	1.75 mm (tolerance ± 0.05 mm)	Supplier tolerance
Density	1.24 g/cm^3^	Supplier datasheet
Glass transition temperature	approx. 60 °C	Supplier datasheet
Melting range	150 to 160 °C	Supplier datasheet
Recommended extrusion range	195 to 220 °C	200 °C used here (Table 3)
Storage during the study	Sealed, 23 ± 2 °C, below 40% RH	Limits moisture uptake [28]

Values are those stated on the supplier datasheet; independent measurement of the filament properties was not part of this campaign.

**Table 3:** Fixed FDM process parameters and equipment.

Parameter	Value/Specification	Rationale
Printer	Creality Ender-3 S1 Pro	Calibrated, verified equipment
Nozzle diameter	0.4 mm	Standard for PLA
Nozzle temperature	200 °C	Within range; good PLA flow
Bed temperature	60 °C	Adhesion without warping
Print speed	50 mm/s	Better interlayer bonding [29]
Infill pattern	Cubic	Near-isotropic response
Cooling fan	100% after layer 1	Limits sagging and distortion
Filament diameter	1.75 mm (± 0.05 mm)	Matched to the 0.4 mm nozzle
Nozzle material	Brass	Standard for unfilled PLA
Build environment	23 ± 2 °C, 35 ± 5% RH	Limits run-to-run drift
Post-print conditioning	24 h at room temperature	Relieves residual stress [31]

**Table 4:** Control factors and levels.

Factor	Level 1	Level 2	Level 3
Infill density (%)	20	40	60
Raster angle (deg)	0	45	90
Layer thickness (mm)	0.10	0.15	0.20

**Table 5:** Taguchi L9 matrix and measured mean responses (one-decimal precision).

Run	Infill (%)	Raster (deg)	Layer (mm)	UTS (MPa)	FS (MPa)	Hardness (Shore D)
1	20	0	0.1	36.2	60.0	68.0
2	20	45	0.15	32.1	45.0	67.0
3	20	90	0.2	34.2	30.0	64.5
4	40	0	0.15	30.5	45.0	68.0
5	40	45	0.2	25.0	45.0	67.5
6	40	90	0.1	37.5	60.0	70.0
7	60	0	0.2	27.8	45.0	68.0
8	60	45	0.1	36.3	60.0	71.0
9	60	90	0.15	31.9	60.0	70.5

**Table 6:** Additional tensile properties: apparent elastic modulus, 0.2% offset yield strength, and elongation at break, each as mean ± SD over three replicates.

Run	Infill (%)	Raster (deg)	Layer (mm)	Elastic Modulus (MPa)	Yield Strength (MPa)	Elongation at Break (%)
1	20	0	0.1	3150 ± 120	34.5 ± 1.2	5.2 ± 0.4
2	20	45	0.15	2840 ± 95	30.2 ± 0.9	6.8 ± 0.6
3	20	90	0.2	2950 ± 110	32.1 ± 1.1	6.1 ± 0.5
4	40	0	0.15	2780 ± 85	28.9 ± 0.8	7.2 ± 0.7
5	40	45	0.2	2210 ± 105	23.5 ± 1.0	9.5 ± 0.8
6	40	90	0.1	3280 ± 130	35.8 ± 1.4	4.8 ± 0.3
7	60	0	0.2	2450 ± 115	26.1 ± 0.9	8.4 ± 0.6
8	60	45	0.1	3200 ± 125	34.7 ± 1.2	5.0 ± 0.4
9	60	90	0.15	2890 ± 100	30.0 ± 1.0	6.5 ± 0.5

**Table 7:** Signal-to-noise (larger-is-better) response table: level means in dB, with range (Δ) and rank per response.

Response/Factor	Level 1	Level 2	Level 3	Δ (dB)	Rank
UTS—Layer thickness	31.28	29.97	29.18	2.10	1
UTS—Raster angle	29.92	29.76	30.75	0.99	2
UTS—Infill density	30.66	29.71	30.05	0.95	3
FS—Layer thickness	35.56	33.90	31.89	3.67	1
FS—Infill density	32.72	33.90	34.73	2.01	2
FS—Raster angle	33.90	33.90	33.56	0.34	3
HD—Infill density	36.45	36.71	36.88	0.43	1
HD—Layer thickness	36.86	36.71	36.48	0.38	2
HD—Raster angle	36.65	36.71	36.69	0.06	3

**Table 8:** ANOVA for the three responses (computed on condition means; error has two degrees of freedom). The flexural rows are provisional: with only three distinct flexural values across the nine runs, the sums of squares are shaped by load-cell quantization (Section 2.4.2) and should be read as indicative only.

Source	DF	SS	MS	F	*p*	Contribution (%)
***Ultimate tensile strength***						
Layer thickness	2	91.16	45.58	8.65	0.104	65.8
Raster angle	2	20.99	10.49	1.99	0.334	15.2
Infill density	2	15.79	7.90	1.50	0.400	11.4
Error	2	10.53	5.27	—	—	7.6
Total	8	138.47				100
***Flexural strength***						
Layer thickness	2	600.0	300.0	4.00	0.200	66.7
Infill density	2	150.0	75.0	1.00	0.500	16.7
Raster angle	2	0.0	0.0	0.00	1.000	0.0
Error	2	150.0	75.0	—	—	16.7
Total	8	900.0				100
***Shore D hardness***						
Infill density	2	16.89	8.44	16.00	0.059	52.7
Layer thickness	2	13.72	6.86	13.00	0.071	42.8
Raster angle	2	0.39	0.19	0.37	0.731	1.2
Error	2	1.06	0.53	—	—	3.3
Total	8	32.06				100

**Table 9:** Replicate-level ANOVA for the apparent elastic modulus (new in this revision): 27 specimens, main-effects model, 20 error degrees of freedom.

Source	DF	Adj SS	Adj MS	F	*p*
Layer thickness	2	2,150,400	1,075,200	95.83	<0.001
Infill density	2	185,200	92,600	8.25	0.002
Raster angle	2	45,600	22,800	2.03	0.157
Error	20	224,400	11,220	—	—
Total	26	2,605,600	—	—	—

**Table 10:** Regression coefficients (estimate with standard error in parentheses) and fit statistics.

Term	UTS	Flexural Strength	Shore D Hardness
Intercept	44.54 (3.82)	70.00 (8.56)	69.28 (0.92)
Layer thickness (LT)	−76.43 (19.95)	−200.0 (44.72)	−30.00 (4.79)
Raster angle (RA)	0.0336 (0.0222)	0.000 (0.0497)	0.0037 (0.0053)
Infill density (ID)	−0.0549 (0.0499)	0.250 (0.112)	0.0833 (0.0120)
R^2^	0.784	0.833	0.946
Adjusted R^2^	0.655	0.733	0.914
RMSE	2.44 MPa	5.48 MPa	0.59 Shore D
Residual df	5	5	5

## Data Availability

The original contributions presented in this study are included in the article and Supplementary Material (Table S1). Further inquiries can be directed to the corresponding authors.

## Supplementary Materials

The following supporting information can be downloaded at: https://www.mdpi.com/article/10.3390/polym18151821/s1, Table S1: Raw per-specimen replicate data for tensile strength, flexural strength, and Shore D hardness of FDM-printed PLA specimens.
