# Supplementary material for: Multi-Objective Optimization of the Mechanical Properties of 3D-Printed PLA: An Integrated Taguchi and NSGA-II Approach
Source: Polymers (Basel). 2026 Jul 25;18(15):1821. doi: 10.3390/polym18151821 (PMC13467678; doi:10.3390/polym18151821)
Supplement: Supplementary file 1 [file polymers-18-01821-s001.zip › polymers-4419422-supplementary.pdf]

Supplementary Materials

Table S1. Raw per-specimen replicate data for tensile strength, flexural strength, and Shore D hardness of FDM-printed PLA specimens.

Condition (Run)	Infill Density (%)	Raster Angle (deg)	Layer Thickness (mm)	Specimen ID	Test Type	Measured Value	Unit
1	20	0	0.1	T-R1-1	Tensile (UTS)	38.75	MPa
1	20	0	0.1	F-R1-1	Flexural Strength	45.65	MPa
1	20	0	0.1	H-R1-1	Shore D Hardness	67.6	Shore D
1	20	0	0.1	T-R1-2	Tensile (UTS)	40.28	MPa
1	20	0	0.1	F-R1-2	Flexural Strength	45.41	MPa
1	20	0	0.1	H-R1-2	Shore D Hardness	66.8	Shore D
1	20	0	0.1	T-R1-3	Tensile (UTS)	40.37	MPa
1	20	0	0.1	F-R1-3	Flexural Strength	47.92	MPa
1	20	0	0.1	H-R1-3	Shore D Hardness	66.5	Shore D
2	20	45	0.2	T-R2-1	Tensile (UTS)	35.81	MPa
2	20	45	0.2	F-R2-1	Flexural Strength	39.84	MPa
2	20	45	0.2	H-R2-1	Shore D Hardness	65.5	Shore D
2	20	45	0.2	T-R2-2	Tensile (UTS)	35.36	MPa
2	20	45	0.2	F-R2-2	Flexural Strength	36.22	MPa
2	20	45	0.2	H-R2-2	Shore D Hardness	64.3	Shore D
2	20	45	0.2	T-R2-3	Tensile (UTS)	34.16	MPa
2	20	45	0.2	F-R2-3	Flexural Strength	38.47	MPa
2	20	45	0.2	H-R2-3	Shore D Hardness	66.3	Shore D
3	20	90	0.3	T-R3-1	Tensile (UTS)	30.64	MPa
3	20	90	0.3	F-R3-1	Flexural Strength	32.47	MPa

3	20	90	0.3	H-R3-1	Shore D Hardness	66.5	Shore D
3	20	90	0.3	T-R3-2	Tensile (UTS)	31.66	MPa
3	20	90	0.3	F-R3-2	Flexural Strength	36.17	MPa
3	20	90	0.3	H-R3-2	Shore D Hardness	63.6	Shore D
3	20	90	0.3	T-R3-3	Tensile (UTS)	31.18	MPa
3	20	90	0.3	F-R3-3	Flexural Strength	36.28	MPa
3	20	90	0.3	H-R3-3	Shore D Hardness	63.8	Shore D
4	40	0	0.3	T-R4-1	Tensile (UTS)	30.56	MPa
4	40	0	0.3	F-R4-1	Flexural Strength	40.5	MPa
4	40	0	0.3	H-R4-1	Shore D Hardness	66.7	Shore D
4	40	0	0.3	T-R4-2	Tensile (UTS)	29.1	MPa
4	40	0	0.3	F-R4-2	Flexural Strength	46.63	MPa
4	40	0	0.3	H-R4-2	Shore D Hardness	67.0	Shore D
4	40	0	0.3	T-R4-3	Tensile (UTS)	28.41	MPa
4	40	0	0.3	F-R4-3	Flexural Strength	44.06	MPa
4	40	0	0.3	H-R4-3	Shore D Hardness	65.8	Shore D
5	40	45	0.2	T-R5-1	Tensile (UTS)	25.31	MPa
5	40	45	0.2	F-R5-1	Flexural Strength	42.1	MPa
5	40	45	0.2	H-R5-1	Shore D Hardness	66.7	Shore D
5	40	45	0.2	T-R5-2	Tensile (UTS)	25.3	MPa
5	40	45	0.2	F-R5-2	Flexural Strength	48.85	MPa
5	40	45	0.2	H-R5-2	Shore D Hardness	68.2	Shore D
5	40	45	0.2	T-R5-3	Tensile (UTS)	24.83	MPa
5	40	45	0.2	F-R5-3	Flexural Strength	46.25	MPa

5	40	45	0.2	H-R5-3	Shore D Hardness	66.5	Shore D
6	40	90	0.1	T-R6-1	Tensile (UTS)	34.92	MPa
6	40	90	0.1	F-R6-1	Flexural Strength	50.85	MPa
6	40	90	0.1	H-R6-1	Shore D Hardness	70.1	Shore D
6	40	90	0.1	T-R6-2	Tensile (UTS)	36.52	MPa
6	40	90	0.1	F-R6-2	Flexural Strength	47.59	MPa
6	40	90	0.1	H-R6-2	Shore D Hardness	69.3	Shore D
6	40	90	0.1	T-R6-3	Tensile (UTS)	35.42	MPa
6	40	90	0.1	F-R6-3	Flexural Strength	50.31	MPa
6	40	90	0.1	H-R6-3	Shore D Hardness	69.6	Shore D
7	60	0	0.2	T-R7-1	Tensile (UTS)	32.55	MPa
7	60	0	0.2	F-R7-1	Flexural Strength	55.33	MPa
7	60	0	0.2	H-R7-1	Shore D Hardness	69.2	Shore D
7	60	0	0.2	T-R7-2	Tensile (UTS)	30.54	MPa
7	60	0	0.2	F-R7-2	Flexural Strength	53.83	MPa
7	60	0	0.2	H-R7-2	Shore D Hardness	71.0	Shore D
7	60	0	0.2	T-R7-3	Tensile (UTS)	30.28	MPa
7	60	0	0.2	F-R7-3	Flexural Strength	52.54	MPa
7	60	0	0.2	H-R7-3	Shore D Hardness	68.9	Shore D
8	60	45	0.1	T-R8-1	Tensile (UTS)	32.21	MPa
8	60	45	0.1	F-R8-1	Flexural Strength	60.03	MPa
8	60	45	0.1	H-R8-1	Shore D Hardness	72.4	Shore D
8	60	45	0.1	T-R8-2	Tensile (UTS)	33.89	MPa
8	60	45	0.1	F-R8-2	Flexural Strength	60.51	MPa

8	60	45	0.1	H-R8-2	Shore D Hardness	71.4	Shore D
8	60	45	0.1	T-R8-3	Tensile (UTS)	33.03	MPa
8	60	45	0.1	F-R8-3	Flexural Strength	58.9	MPa
8	60	45	0.1	H-R8-3	Shore D Hardness	72.5	Shore D
9	60	90	0.3	T-R9-1	Tensile (UTS)	27.95	MPa
9	60	90	0.3	F-R9-1	Flexural Strength	51.91	MPa
9	60	90	0.3	H-R9-1	Shore D Hardness	66.4	Shore D
9	60	90	0.3	T-R9-2	Tensile (UTS)	29.23	MPa
9	60	90	0.3	F-R9-2	Flexural Strength	48.22	MPa
9	60	90	0.3	H-R9-2	Shore D Hardness	68.7	Shore D
9	60	90	0.3	T-R9-3	Tensile (UTS)	28.14	MPa
9	60	90	0.3	F-R9-3	Flexural Strength	43.03	MPa
9	60	90	0.3	H-R9-3	Shore D Hardness	68.8	Shore D
